# ﻿Chromosomal polymorphism in natural populations of *Chironomus* sp. prope *agilis* Kiknadze, Siirin, Filippova et al., 1991 (Diptera, Chironomidae)

**DOI:** 10.3897/compcytogen.v16.i4.95659

**Published:** 2022-12-16

**Authors:** Veronika V. Golygina

**Affiliations:** 1 The Federal Research Center Institute of Cytology and Genetics of Siberian Branch of the Russian Academy of Science, Prospect academika Lavrentieva 10, Novosibirsk, 630090 Russia The Federal Research Center Institute of Cytology and Genetics of Siberian Branch of the Russian Academy of Science Novosibirsk Russia

**Keywords:** banding sequence, *Ch.plumosus* group, inversion, karyological analysis, karyotype, polythene chromosome, sibling species

## Abstract

Species Chironomussp.propeagilis Kiknadze, Siirin, Filippova et al., 1991 belongs to the *Ch.plumosus* group of sibling species. It was described on the basis of its karyotype and analysis of isozymes from one population in the Urals but since then no quantitative data on chromosomal polymorphism of this species have been published. The goal of this study is to broaden our knowledge of the chromosomal polymorphism and distribution of the Chironomussp.propeagilis, which, along with the data on chromosomal polymorphism of other species from the *Ch.plumosus* group, can give us a better understanding of the connection between chromosomal polymorphism and ecological conditions of habitats. The specimens of Chironomussp.propeagilis were found only in 8 natural populations from the Urals, Western Siberia and Kazakhstan, which allows us to conclude that the species range of Chironomussp.propeagilis is not as wide as for most other species from *Ch.plumosus* group. An analysis of chromosomal polymorphism in these 8 natural populations of Chironomussp.propeagilis has been performed. All of the studied populations were either monomorphic or showed very low level of chromosomal polymorphism, with 4.4–8.7% of heterozygous specimens per population and 0.04–0.08 heterozygotic inversion per larvae. The total number of banding sequences found in the banding sequence pool of Chironomussp.propeagilis is 10. The mapping of banding sequence p’ag2B3 is presented for the first time. Besides inversions, one reciprocal translocation was found in a population from Kazakhstan, B-chromosome was found in one population from the Urals region of Russia, and heterozygosity of the level of expression of Balbiany rings in arm G was observed in several studied populations.

## ﻿Introduction

The species Chironomussp.propeagilis is one of the rarest species in the *Ch.plumosus* group of sibling species. It was first described in 1991 from the lake near Yurgamish settlement in the Urals based on its karyotype and is closest to *Ch.agilis* Shobanov et Djomin, 1988 (Kiknadze et al. 1991a). These two species differ mainly by the size of centromeric heterochromatin – medium in *Ch.agilis* but very large in Chironomussp.propeagilis – and the dominant banding sequence in one chromosomal arm. The status of Chironomussp.propeagilis as a separate species in the *Ch.plumosus* group of sibling species was also confirmed by isozyme analysis, which showed that genetic distances between this species and other species from the *Ch.plumosus* group correspond to values typically observed for genetic distances between sibling species in chironomids (Gunderina et al. 1988; Filippova et al. 1989, 1990).

Since its first description, no information about chromosomal polymorphism of Chironomussp.propeagilis was published until the recent work of Kiknadze and coauthors (2016), where only information on the banding sequence pool (photos and mapping of banding sequences) of the species was presented with no quantitative data on polymorphism in studied populations. Yet the knowledge of the patterns of chromosomal polymorphism in natural populations is essential for gaining a better understanding of the connection between chromosomal polymorphism and ecological conditions of habitats, and *Ch.plumosus* group of sibling species present a great model for such studies.

Thus, the purpose of this paper is to present new data on chromosomal polymorphism in populations of Chironomussp.propeagilis from the Russian Federation and Kazakhstan.

## ﻿Material and methods

The VI instar larvae from 8 natural populations from Russia (the Urals and Siberia) and Kazakhstan were used for polytene chromosome slide preparation. Data on collection sites is presented in Table 1.

The larvae were fixed with 3:1 *v/v* of 96% ethanol and glacial acetic acid and stored at –20 °C. Polytene chromosome squashes were prepared by the routine aceto-orcein method (Keyl and Keyl 1959; Kiknadze et al. 1991b). Chromosomal mapping of arms A, C, D, E, and F was done using the mapping system created by Keyl (1962) and Dévai et al. (1989), with *Ch.piger* Strenzke, 1959 as the standard karyotype. Mapping of arm B was done according to the Maximova mapping system (Maximova 1976), improved by Schobanov (1994), with *Ch.plumosus* chromosomes as the standard.

Each banding sequence is given a short designation as follows: three-letter abbreviation of the species name (ag2 as in the first description, the species was named *Ch.agilis* 2 and the abbreviation ag2 was used in all subsequent works) followed by the name of the arm and the serial number of banding sequence in this arm (according to the order of its discovery), and prefixed by a letter indicating its geographical distribution in the genus *Chironomus* (p’ for Palearctic sequences or h’ for Holarctic sequences). For example, h’ag2E1 means that while Ch.sp.propeagilis itself is a Palearctic species, this banding sequence is identical to banding sequences of some other species and was found both in the Palearctic and the Nearctic and thus is a Holarctic banding sequence.

Statistical analysis was done using the program PHYLIP (https://evolution.genetics.washington.edu/phylip.html).

The following equipment of the Centre of Microscopical analysis of biological objects SB RAS in the Institute of Cytology and Genetics (Novosibirsk) was used for this work: microscope “Axioskop” 2 Plus, CCD-camera AxioCam HRc, software package AxioVision 4 (Zeiss, Germany).

## ﻿Results and discussion

**Figure 1.**
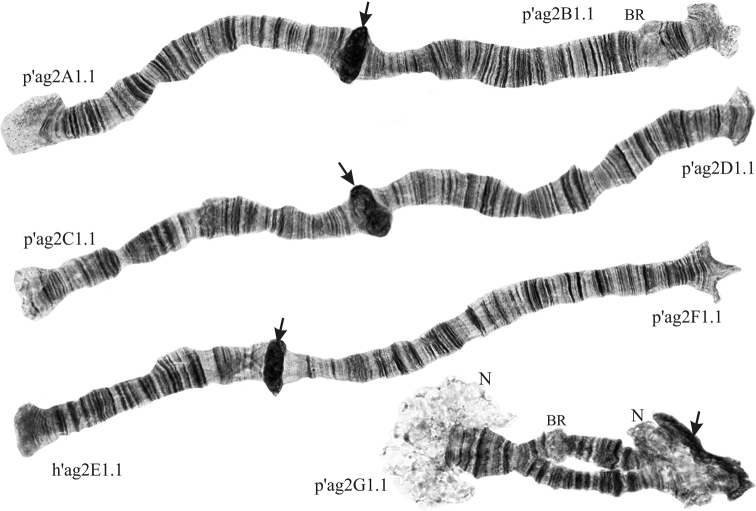
Karyotype of Chironomussp.propeagilis. Centromeric regions are designated by arrows. N – nucleolus, BR – Balbiani Ring.

As all other members of the *Ch.plumosus* group of sibling species, Chironomussp.propeagilis belongs to the “thummi” cytocomplex with a haploid number of chromosomes n = 4 and an arm combination AB CD EF G. The chromosomes I (AB) and II (CD) are metacentric, III (EF) is submetacentric, and IV (G) is telocentric (Fig. 1). There are two nucleoli in Chironomussp.propeagilis karyotype; both are situated on arm G – one on the centromeric end of the arm, the other on their opposite end near the telomere. Homologues of arm G are paired but often unconjugated at the ends in nucleolus regions. Centromeric regions are very large, which, along with two nucleoli on arm G, clearly differentiates this species from the rest of its siblings. There are three Balbiani Rings (BR) in the karyotype of *Ch.agilis*: two are situated on the arm G (usually only the one in the center of the arm is visible as the other one is often masked by the nucleolus), and the third one is on the arm B.

**Figure 2.**
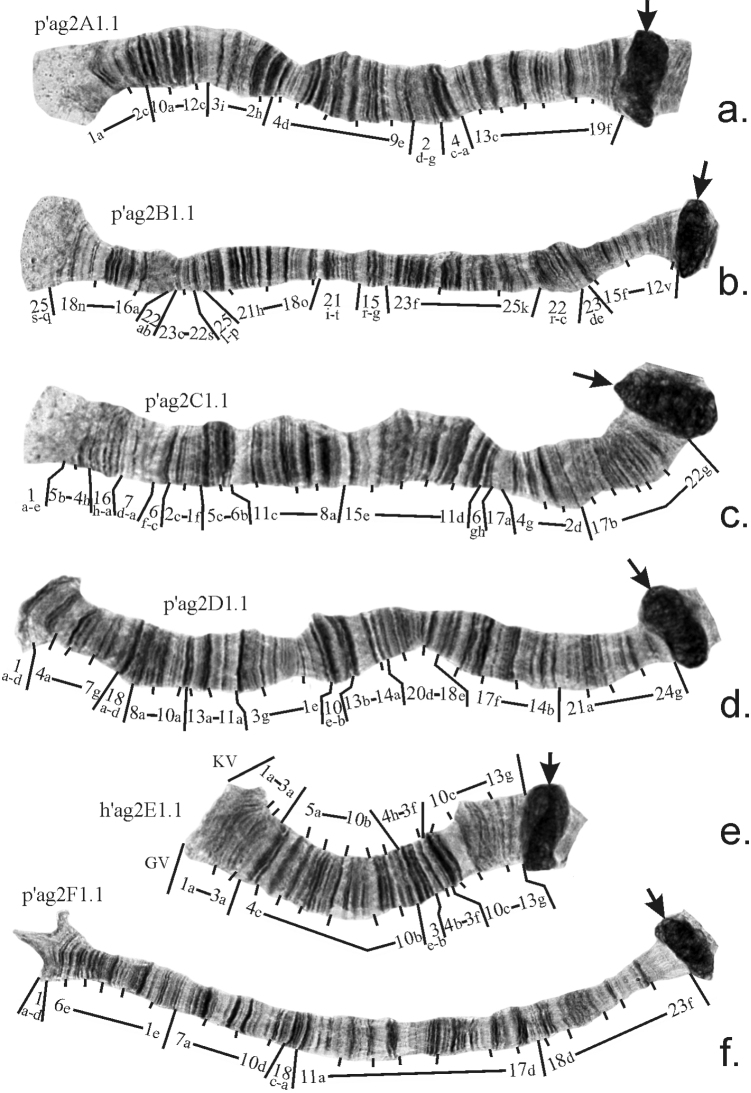
Mapping of main banding sequences in arms **A–F** of Chiroomussp.propeagilis. Centromeric regions are designated by arrows. KV – version of mapping in arm E according to Keyl (1962), GV – version of mapping in arm E according to Golygina and Kiknadze (2018).

The revision of the mapping of main banding sequences in arms A, B, C, D, E, and F was presented by Golygina and Kiknadze previously (2008, 2012, 2018). A revised mapping of these banding sequences is shown in Figure 2. For arm E, two versions of the mapping are presented (Fig. 2e).The first one is done according to how Chironomussp.propeagilis banding sequence should be mapped if mapping of *Ch.plumosus* (the reference species for mapping of all *Ch.plumosus* group sibling species) made by Keyl (1962) is considered to be correct (marked as KV). The second one is done according to the revised mapping of *Ch.plumosus* made by Golygina and Kiknadze (2018) (marked as GV).

As was mentioned above, Chironomussp.propeagilis is a very rare species. Among over 200 populations of chironomids studied from Eurasia by us during the last 30 years, this species was found only in 8 (Table 1), and only in 5 of them – KUR-YU, NSK-IT, ALT-GT, ALT-GR (Russia) and KAZ-KA (Kazakhstan) – we found enough larvae to perform quantitative analysis of inversion polymorphism.

**Table 1.** Collection sites.

Collection place	Abbreviation	Collection date	Geographic coordinates	Number of larvae
**RUSSIA**				
**The Urals**				
**Kurgan region**				
Lake near Yurgamish settlement	KUR-YU	27.02.1990	55°20'54.3"N, 64°28'02.9"E	80
**Western Siberia**				
**Novosibirsk region**				
Itkul Lake	NSK-IT	15.04.1993	55°04'27.3"N, 81°01'53.2"E	12
**Altai territory**				
Gor’koe Lake, Tumentsevo district	ALT-GT	13.05.1993	53°26'48.9"N, 81°22'47.0"E	11
Gor’koe-Peresheechnoe Lake, Egorievo district	ALT-GP	16.05.1994	51°47'04.2"N, 80°50'22.3"E	1
Gor’koe Lake, Rubtsovks district	ALT-GR	04.04.1993 17.05.1993 10.09.1993	51°37'25.3"N, 81°13'23.9"E	23
Tepliy Klyuch Lake near Yarovoe town, Slavgorod district	ALT-TK	04.07.2001	52°19'11.9"N, 83°11'24.2"E	2
Travyanoe Lake, Oskolkovo settlement	ALT-TR	08.05.1994	52°19'11.9"N, 83°11'24.2"E	1
**KAZAKHSTAN**				
Karasor Lake, mouth of river Tundik	KAZ-KA	23.09.1995	53°00'13.5"N, 70°50'15.7"E	64

The main banding sequences of Chironomussp.propeagilis in all arms except arms B and C are identical to the main banding sequences of *Ch.agilis* (Table 2, Fig. 2). The banding sequence p’ag2B1 is identical to p’agiB2 – the alternative banding sequence of *Ch.agilis*, which is prevalent in all studied populations of this species from Siberia and the Far East. The arm C of Chironomussp.propeagilis differs from the main banding sequence of *Ch.agilis* p’agiC1 by a large complex inversion (Table 2, Fig. 2) Previously, p’ag2C1 was considered to be unique to the species, but recent data on chromosomal polymorphism of *Ch.agilis* (Golygina and Ermolaeva 2021) have shown that a banding sequence identical to p’ag2C1 does exist in the banding sequence pool of *Ch.agilis* (p’agiC2), although up to now it has been found only once. Thus, none of the main banding sequences of Chironomussp.propeagilis are unique to the species, so karyologically it is closer to *Ch.agilis* that was presumed previously. The main feature that differentiates the karyotypes of these two species is the size of their centromeric regions.

**Table 2.** Mapping of banding sequences of Chiornonomussp.propeagilis.

Designation of banding sequence	Mapping of banding sequence
p’ag2A1	1a-2c 10a-12c 3i-2h 4d-9e 2d-g 4c-a 13a-19f C
p’ag2A2	1a-2c 10a-12c 3i-2h 4d-7b 4bc 2g-d 9e-7c 4a 13a-19f C
p’ag2B1	25s-q 18n-16a 22ab 23c-22s 25l-p 21h-18o 21i-t 15r-g 23f-25k 22r-c 23de 15f-12v C
p’ag2B2	25s-q 18n-16a 22ab 23c-22s 25l-p 21h-18o 21i-t 15r-o 23z-f 15g-n 24a-25k 22r-c 23de 15f-12v C
p’ag2B3	25s-q 18n-16a 22ab 23c-22s 25l-p 21h-18o 21i-t 15r-g 23f-24s 15a-f 23ed 22c-r
	14r-12v C
p’ag2C1	1a-e 5b-4h 16h-a 7d-a 6f-c 2c-1f 5c-6b 11c-8a 15e-11d 6gh 17a 4g-2d 17b-22g C
p’ag2D1	11a-d 4a-7g 18a-d 8a-10a 13a-11a 3g-1e 10e-b 13b-14a 20d-18e 17f-14b 21a-24g C
h’ag2E1	1a-3e 5a-10b 4h-3f 10c-13g C †
	1a-3a 4c-10b 3e-b 4b-3f 10c-13g C‡
h’ag2F1	1a-d 6e-1e 7a-10d 18c-a 11a-17d 18d-23f C

† - mapped according to Keyl (1962). ‡ - revised mapping according to Golygina and Kiknadze (2018).

**Figure 3.**
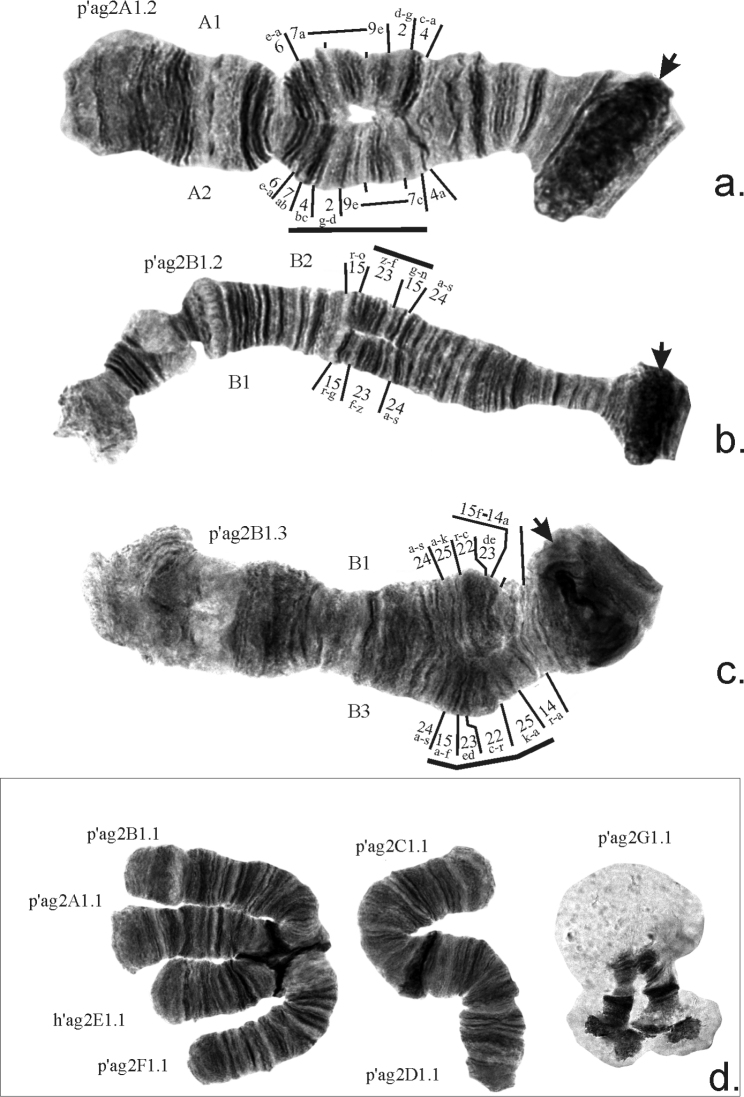
Chromosomal polymorphism found in populations of *Ch.agilis***a–c** inversions in chromosome I (AB) **d** reciprocal translocation. Centromeric regions are designated by arrows. Brackets show regions of inversions.

Inversion polymorphism was observed only in arms of chromosome I (AB), and among three inversions found, only banding sequence p’ag2A2 occurred in several populations with low frequency, the other two – p’ag2B2 and p’ag2B3 – were unique (Tables 3, 4). All three inversions were quite short, thus they could not form standard inversion loops and they are seen as unpaired regions (Fig. 3) and are easy to miss if a researcher didn’t carefully inspect the entire banding pattern of an arm. Mapping of banding sequence p’ag2B3 is presented for the first time (Table 2). Thus, in total, the banding sequence pool of Chironomussp.propeagilis at present consists of 10 banding sequences.

**Table 3.** Frequencies of genotypic combinations of banding sequences and general characteristics of chromosomal polymorphism in populations of Chironomussp.propeagilis.

Genotypic combination	Russia							Kazakhstan
	KUR-YU^§^	NSK-IT	ALT-GT	ALT-GP	ALT-GR	ALT-TK	ALT-TR	KAZ-KA
	80^|^	12	11	1	23	2	1	64
p’ag2A1.1	**0.962**	**0.917**	**1**	1	**0.913**	1	1	**0.984**
p’ag2A1.2	**0.038**	**0.083**	**0**	0	**0.087**	0	0	**0.016**
p’ag2B1.1	**0.987**	**1**	**0**	1	**1**	1	1	**0.984**
p’ag2B1.2	**0.013**	**0**	**1**	0	**0**	0	0	**0**
p’ag2B1.3	**0**	**0**	**0**	0	**0**	0	0	**0.016**
p’ag2C1.1	**1**	**1**	**1**	1	**1**	1	1	**1**
p’ag2D1.1	**1**	**1**	**1**	1	**1**	1	1	**1**
h’ag2E1.1	**1**	**1**	**1**	1	**1**	1	1	**1**
p’agiF1.1	**1**	**1**	**1**	1	**1**	1	1	**1**
p’agiG1.1	**1**	**1**	**1**	1	**1**	1	1	**1**
Percentage of larvae with B-chromosome	**1.3**	**0**	**0**	0	**0**	0	0	**0**
Percentage of larvae showing heterozygocity in the development of BR in arm G	**25**	**0**	**54**	0	**22**	0	0	not studied due to the bad banding structure of arm G
Percentage of larvae with undeveloped BR in arm G	**13**	**0**	**18**	0	**4**	0	0	-
Number of banding sequences	**9**	**8**	**7**	7	**8**	7	7	**9**
Number of genotypic combinations of banding sequences	**9**	**8**	**7**	7	**8**	7	7	**9**
% of heterozygous larvae	**3.75**	**8.3**	**0**	0	**8.7**	0	0	**3.1**
Number of heterozygous inversions per larvae	**0.04**	**0.08**	**0**	0	**0.09**	0	0	**0.03**

^§^ - populations highlighted with bold were used for quantitative analysis of chromosomal polymorphism. ^|^ - number of larvae studied.

**Table 4.** Frequencies of banding sequences in populations of Chironomussp.propeagilis. ^¶^

Banding sequnce	Russia				Kazakhstan
	KUR-YU	NSK-IT	ALT-GT	ALT-GR	KAZ-KA
	80^#^	12	11	23	64
p’ag2A1	0.981	0.959	1	0.957	0.992
p’ag2A2	0.019	0.041	0	0.043	0.008
p’ag2B1	0.994	1	0	1	0.992
p’ag2B2	0.006	0	1	0	0
p’ag2B3	0	0	0	0	0.008
p’ag2C1	1	1	1	1	1
p’ag2D1	1	1	1	1	1
h’ag2E1	1	1	1	1	1
p’agiF1	1	1	1	1	1
p’agiG1	1	1	1	1	1

^¶^ - only populations with enough larva for quantitative analysis (more than 10 specimens) are included into this table. ^#^ - number of larvae studied.

Besides inversions, one reciprocal translocation was found in a population from Kazakhstan (Fig. 3, d). Heterozygosity of development of BR and underdevelopment of BR on both homologs in the center of the arm G was observed in populations from the Ural and Altai regions (Fig. 1, Table 3). The genomic polymorphism in the form of an additional B-chromosome was found in one larva from the KUR-YU population from the Urals (Table 3).

Thus, Chironomussp.propeagilis can be considered as having a very low level of polymorphism. Among all studied species from the plumosus group, with the exception of *Chironomusbonus* Shilova et Dzhvarsheishvili, 1974, which also has only a few studied populations, Chironomussp.propeagilis is the most monomorphic. Cytogenetic distances between populations varied from 0 to 0.008.

Although there are currently no hard data on the water characteristics in the waterbodies where Chironomussp.propeagilis was recorded (such as salinity, ion content etc.), it is possible to speculate that this species is likely adapted to life in somewhat saline waters. We suggest this conclusion as most lakes where it was found can be categorized as saline (the name ‘Gor’koe’ means ‘bitter’ and is given in the Altai region to saline lakes, and Karasor Lake in Kazakhstan is also a confirmed saline lake). It is possible that the low level of chromosomal polymorphism, as well as the rarity of these species, can also be attributed to its preference in habitats, although in order to make a firm conclusion on this matter, more studies of the species are required. At present, the species range of the Chironomussp.propeagilis can be defined as covering the Urals, south of Western Siberia and Northern Kazakhstan.
